# Metachronous occurrence of main-duct intraductal papillary mucinous neoplasm (IPMN) and adenocarcinoma in a chronic pancreatitis patient: A case report

**DOI:** 10.1097/MD.0000000000028770

**Published:** 2022-02-11

**Authors:** Keum Nahn Jee

**Affiliations:** Department of Radiology, Dankook University Hospital, Cheonan, South Korea.

**Keywords:** case report, chronic pancreatitis, intraductal papillary mucinous neoplasm, metachronous neoplasm, pancreatic ductal adenocarcinoma

## Abstract

**Rationale:**

Chronic pancreatitis (CP) is a risk factor for developing pancreatic ductal adenocarcinoma (PDAC). In addition, a patient with partial pancreatectomy for intraductal papillary mucinous neoplasm (IPMN) can also lead to PDAC. In contrast, IPMN is a distinct disease entity, independent of CP, and there have been few reports that CP is the cause of IPMN. To the best of our knowledge, this is the first clinical case report of the metachronous occurrence of main-duct IPMN and PDAC with a 9 and half-year interval in a patient with chronic alcoholic pancreatitis.

**Patient concerns:**

A 50-year-old man with a long medical history of recurrent alcoholic pancreatitis and hepatitis over a decade was diagnosed with another episode of acute pancreatitis based on laboratory findings and clinical symptoms. The patient underwent pylorus-preserving pancreaticoduodenectomy (PPPD) for a small nodular lesion in the main duct of the pancreatic head and was diagnosed with main-duct IPMN low-grade dysplasia and associated fibrosing CP. Nine and a half years later, a 59-year-old man lost 7 kg over 3 months and was diagnosed with new-onset diabetes mellitus.

**Diagnosis:**

The patient was diagnosed with metachronous, well-differentiated PDAC with concomitant CP.

**Interventions:**

The patient underwent radical antegrade modular pancreatosplenectomy (RAMPS) for a small nodular mass in the remnant pancreas.

**Outcomes:**

The patient was healthy for 44 months without evidence of tumor recurrence during clinical follow-up examinations including laboratory findings, tumor marker, and imaging studies.

**Lessons:**

Early diagnosis of metachronous pancreatic neoplasia in a patient with chronic pancreatitis could be made by correlating newly developed clinical symptoms and signs with careful radiological examinations.

## 1 Introduction

Chronic pancreatitis (CP) is characterized by continuous inflammatory fibrosis of the pancreatic tissue. The most common etiology of CP is heavy alcohol consumption. CP and heavy drinking are recognized risk factors for developing pancreatic ductal adenocarcinoma (PDAC).^[1,2]^ The overall incidence of CP-associated PDAC remains unclear with reports of varying occurrence rates.^[2,3]^ Intraductal papillary mucinous neoplasm (IPMN) is a distinct disease entity independent of CP, and is mucin-producing tumor of the pancreas by the papillary proliferation of atypical mucinous epithelium in the main or branched pancreatic ducts. IPMNs are a heterogeneous group of tumors with a wide range of grades and histological subtypes, diverse prognoses, and multifocal occurrences. Main-duct IPMN is less frequent and has a higher risk of malignant transformation than that of branch-duct IPMNs.^[4,5]^ PDAC can be derived from an IPMN or could develop in the same pancreas apart from the IPMN and even after IPMN resection. Metachronous occurrence of IPMN or pancreatic ductal adenocarcinoma after partial resection of the pancreas due to IPMN has been reported to have a wide range of incidence rates.^[6–8]^ However, to date, few reports support that CP may be the cause of IPMN, except for one study that mentioned CP may be an independent risk factor for developing IPMNs, especially main-duct IPMNs.^[9]^

This is the first clinical case report, in English-written medical literature, about the metachronous occurrence of main-duct IPMN and PDAC with a nine and half-year interval in chronic alcoholic pancreatitis patient.

This study was approved and written informed consent was waived by institutional review board of Dankook University Hospital.

## 2 Case presentation

A 50-year-old man presented with epigastric pain, fever, and chills. At the time, carbohydrate antigen 19-9 (627 U/mL; normal range, 0–37 U/mL) and total bilirubin (1.96 mg/dL; normal range, 0.2–1.2 mg/dL) values were elevated. Elevated amylase and lipase levels and white blood cell counts were also observed, and he was clinically diagnosed with acute pancreatitis.

He had been a heavy drinker for 30 years and was hospitalized several times over a decade for recurrent alcoholic pancreatitis and hepatitis, associated with excessive drinking. Computed tomography (CT) images at those times showed diffuse dilatation of the main pancreatic duct without intraductal focal lesion and peripancreatic fluid collection or pseudocyst.

However, unlike before, at this time of acute pancreatitis, excessive drinking was unclear.

**Figure 1:**
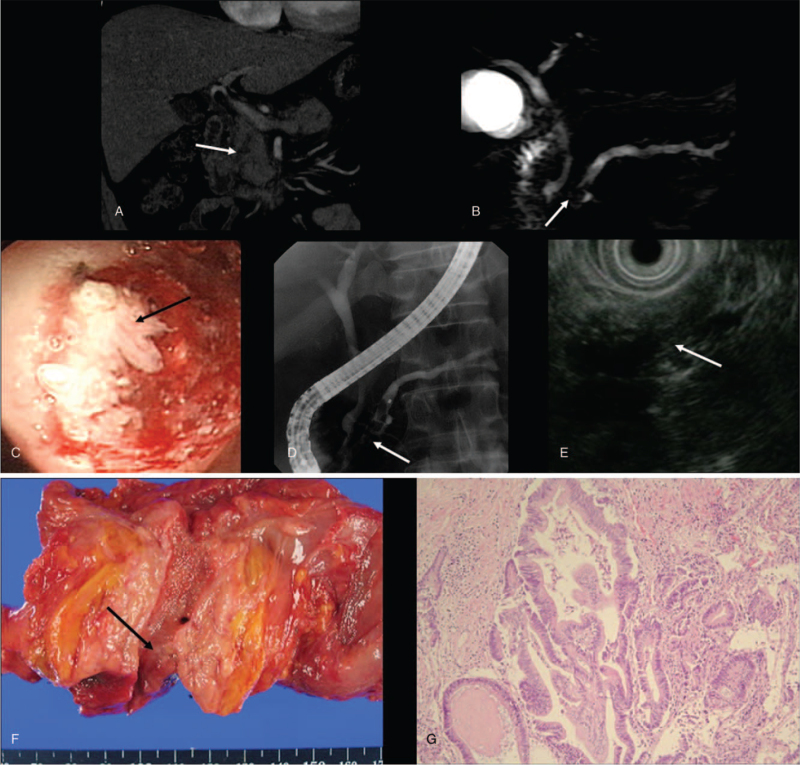
(A) Contrast-enhanced CT image with coronal reformation shows an ill-defined small nodule at the head of dilated main pancreatic duct (arrow). (B) The MRCP image shows an irregularly margined small dark signal intensity nodule (arrow) in the beaded dilated main pancreatic duct. (C) ERCP shows a small papillary lesion (arrow) with surrounding mucin in the head of the main pancreatic duct. (D) ERCP shows a nodular filling defect at head of dilated main duct. (E) EUS shows a small hyperechoic nodule in the dilated main pancreatic duct. (F) Gross pathologic specimen of PPPD shows slight elevated mucosal surface (arrow) at the head portion of the dilated main pancreatic duct. (G) Microscopic histopathology (hematoxylin and eosin, 200 x magnification) shows mild atypical mucinous epithelium with reminiscent of gastric foveolar epithelium suggesting gastric subtype IPMN low-grade dysplasia. CT = computed tomography, ERCP = endoscopic retrograde cholangiopancreatography, EUS = endoscopic ultrasonography, IPMN = intraductal papillary mucinous neoplasm, MRCP = magnetic resonance cholangiopancreatography, PPPD = pylorus-preserving pancreaticoduodenectomy.

Contrast-enhanced CT (CECT) suggested an ill-defined small nodule in the head portion of the dilated main pancreatic duct (Fig. 1A) that newly appeared compared with the CT scan 13 months earlier and associated pancreatic parenchymal swelling, peripancreatic fat strands, and mild fatty liver.

Magnetic resonance cholangiopancreatography (MRCP) showed an irregular margined small nodule (10 mm) at the head of the dilated main pancreatic duct (Fig. 1B). Endoscopic retrograde cholangiopancreatography (ERCP) showed the bulging duodenal papilla with a small amount of mucin expulsion and a focal papillary mucosal surface at the head of the main duct (Fig. 1C and D). Endoscopic ultrasonography (EUS) revealed a slightly hyperechoic small nodule in the dilated main pancreatic duct (Fig. 1E). The patient underwent pylorus-preserving pancreaticoduodenectomy (PPPD). A PPPD specimen revealed a small papillary lesion on the surface of the main duct in the head (Fig. 1F). Histopathology revealed main-duct IPMN low-grade dysplasia without stromal invasion (Fig. 1G) and associated chronic fibrosing pancreatitis.

The patient rarely consumed alcoholic beverages and had no recurrence of pancreatitis or hepatitis or neoplasm during clinical and radiological follow-up for 115 months after PPPD.

However, nine and half years later, the 59-year-old patient lost 7 kg over 3 months and was diagnosed with new-onset diabetes mellitus. At that time, the laboratory test values were within the normal ranges, including amylase, lipase, white blood cell count, C-reactive protein, and CA 19-9, except for elevated fasting blood glucose levels (217 mg/dL; normal range, 55–115 mg/dL).

**Figure 2:**
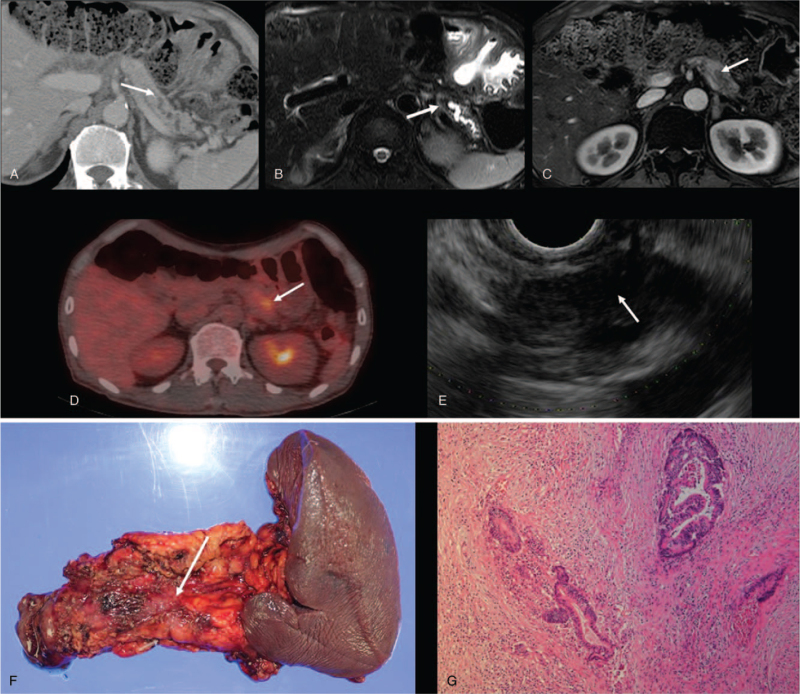
(A) Contrast-enhanced CT image shows a stricture (arrow) at the body with dilated upstream main duct in remnant pancreas. (B) Axial T2-weighted MRI shows a small low-signal-intensity nodule (arrow) at pancreatic body with dilated upstream main duct. (C) Contrast-enhanced arterial-phase T1-weighted MR image shows a small nodule with low signal intensity (arrow) at the body with a dilated upstream main pancreatic duct. (D) ^18^F-FDG PET/CT fusion image shows a focal FDG-avid lesion (arrow) in the pancreatic body. (E) EUS shows an ill-defined hypoechoic small nodule at the obstruction site of the main pancreatic duct. (F) Gross pathologic specimen shows an ill-defined small mass (arrow) in the body of the remnant pancreas. (G) Microscopic histopathology (hematoxylin and eosin, x 200) shows well-differentiated adenocarcinoma. ^18^F-FDG PET/CT =  F-fluorodeoxyglucose (^18^F-FDG) positron emission tomography/computed tomography, CT = computed tomography, EUS = endoscopic ultrasonography, MRI = magnetic resonance imaging.

CECT revealed a stricture in the body with dilation of the upstream main duct in the remnant pancreas (Fig. 2A) that newly appeared compared with the CT scan 3 months earlier. Magnetic resonance imaging (MRI) showed a 1-cm sized small nodule in the pancreatic body with a dilated upstream main duct in the remnant pancreas (Fig. 2B and C). F-fluorodeoxyglucose (^18^F-FDG) positron emission tomography/computed tomography fusion image showing a focal FDG-avid lesion (SUVmax max 3.8) at the body of the remnant pancreas (Fig. 2D).

EUS showed an ill-defined small hypoechoic nodule in the body with a dilated upstream main pancreatic duct (Fig. 2E). EUS-guided fine-needle aspiration biopsy (FNA) suggested malignant cells. Then, the patient underwent radical antegrade modular pancreatosplenectomy (RAMPS) of the remnant pancreas (Fig. 2F).

Histopathology showed well-differentiated PDAC as a small (1.2 cm) infiltrative mass and no metastatic lymph nodes, and concomitant fibrosing CP (Fig. 2G).

He had been healthy for 44 months without evidence of tumor recurrence during clinical follow-up examinations, including laboratory findings, tumor markers, and imaging studies like CT, MRI, and F-fluorodeoxyglucose (^18^F-FDG) positron emission tomography/computed tomography.

## 3 Discussion

### 3.1 Chronic pancreatitis and intraductal papillary mucinous neoplasm

CP and IPMN are completely different disease entities. Several studies have proposed main-duct IPMN could produce chronic obstructive pancreatitis by mucin blockade of pancreatic ducts. Clinical and pathological features suggest that in most cases, IPMN is the cause of CP and not vice versa. In addition, there have been a few reports that IPMNs could be mistaken for CP owing to clinical manifestations and imaging findings similar to those of CP.^[8,10–13]^

However, there has been only one case report of the development of invasive carcinoma with IPMN after partial pancreatic resection due to chronic pancreatitis.^[14]^

Capurso et al.^[9]^ reported that the CP concurrence rate among patients with IPMNs was significantly higher than that in the control group (3.1% vs 0.3%), and a history of CP may be a relevant independent risk factor for IPMN, especially main-duct IPMN.

In contrast, Kamata et al.^[15]^ reported that the risk factors for PDAC, including CP, were not significantly associated with the malignant IPMN like high-grade dysplasia and associated invasive carcinoma.

This patient had recurrent acute pancreatitis related to excessive alcohol consumption for more than a decade, and the associated computed tomography (CT) findings showed acute interstitial edematous pancreatitis with peripancreatic fluid or pseudocyst. CT scan 28 months before diagnosis of main-duct IPMN showed diffuse mild dilatation of the main pancreatic duct without focal lesions suggesting morphological changes in chronic pancreatitis, and there was no mucin expulsion or duct obstruction on the ERCP.

The first surgery, PPPD, revealed main-duct IPMN low-grade dysplasia with associated chronic fibrosing pancreatitis.

### 3.2 Intraductal papillary mucinous neoplasm and pancreatic ductal adenocarcinoma

Patients after a partial pancreatectomy due to IPMN remain at risk for developing metachronous IPMN or concomitant PDAC. However, the recurrent rate of metachronous IPMN following partial pancreatic resection is difficult to determine because of the multifocal occurrence of IPMN and the difficulty in the differentiation of recurrent main-duct IPMN from postoperative anastomotic stricture. So, the recurrence rates of IPMN after partial pancreatectomy due to IPMN were reported to be 0% to 20% and invasive recurrence rates of 2% to 7.8%.^[4,6–8,16,17]^

Patients with IPMN of high-grade dysplasia, pancreatobiliary subtype, resection margin positive previous surgery, and a family history of PDAC have a significantly higher rate of recurrence or metachronous IPMN. Metachronous high-risk lesions, such as high-grade dysplasia or invasive cancer can often develop more than 5 to 10 years after partial pancreatectomy and the cumulative 5-year incidence of concomitant high-risk lesions ranges from 2.2% to 8.8%.^[7,8,17,18]^

The PDAC derived from IPMN and concomitant with IPMN were reported to be significantly smaller at diagnosis and is less invasive than PDAC, independent of IPMN. In addition, after resection of the mainduct IPMN without macroscopic invasive carcinoma, the prognosis is usually favorable.^[4,6,8]^

In this case, PDAC developed in the remnant pancreas 115 months after resection of the main duct IPMN low-grade dysplasia.

### 3.3 Chronic pancreatitis and pancreatic ductal adenocarcinoma

Previous studies, mostly case-control studies, have reported that the relative risk of PDAC in patients with CP varied widely from 2.3 to 18.5.^[2,3]^

In a meta-analysis, they concluded that chronic pancreatitis has a markedly increased risk of PDAC. Five years after the diagnosis, patients with CP have a nearly 8-fold increased risk of PDAC; and the risk remains high for almost 10 years after the diagnosis, and then declines over time.^[19]^

However, another study reported that the risk of PDAC in patients with CP increased over time, approximately 1.8% at 10 years and 4% at 20 years.^[20]^

In this case, more than ten years had passed since the diagnosis of chronic pancreatitis due to recurrent alcoholic pancreatitis at the time of diagnosis of PDAC.

### 3.4 Chronic pancreatitis, intraductal papillary mucinous neoplasm, and pancreatic ductal adenocarcinoma

Both CP and IPMN are risk factors for developing PDAC. KRAS mutation is known to be an important event in IPMN and PDAC carcinogenesis and occurs at a relatively early stage in both the cases. In patients with PDAC, KRAS oncogene mutations have been identified in up to 95% to 100%.^[21]^

In addition, KRAS mutations have been found in patients with CP with a 3-year disease history. But, some chronic pancreatitis patients with KRAS mutations never develop PDAC.^[22,23]^

In this case, the patient had two potential risk factors of IPMN and CP for developing PDAC.

However, he was not examined for KRAS expression.

### 3.5 Clinical features at the diagnosis of intraductal papillary mucinous neoplasm and pancreatic ductal adenocarcinoma

A small papillary lesion with mucin in the main pancreatic duct appeared to cause acute pancreatitis in this patient with a long history of recurrent alcoholic pancreatitis. At that time, elevated CA 19-9 level and atypical cells with mucin on FNA biopsy led to the diagnosis of PPPD.

Elevated CA 19-9 and total bilirubin levels at the time of IPMN diagnosis became normal after the surgery.

For this patient, the diagnosis of new-onset DM made it possible to diagnose it as metachronous neoplasm of PDAC as a small-sized mass at an early stage after PPPD due to main-duct IPMN.

The diagnosed PDAC was 1.2-cm in size and well-differentiated type without lymph node metastasis. And histopathology showed the concomitant fibrosing CP without associated recurrent IPMN.

Although the relationship between main duct IPMN and the development of the PDAC is known, there have been no cases of PDAC occurring 115 months after resection for main-duct IPMN low-grade dysplasia.

To the best of our knowledge, this is the first clinical case report of a metachronous occurrence of main-duct IPMN low-grade dysplasia and well-differentiated PDAC within a nine and half-year interval in a chronic alcoholic pancreatitis patient.

## Acknowledgment

The author thanks the Drs at the surgical and pathological departments for their comments and discussions about the case.

## Author contributions

Case review, study design, investigation, supervision, and writing: Keum Nahn Jee.

**Conceptualization:** Keum Nahn Jee.

**Data curation:** Keum Nahn Jee.

**Formal analysis:** Keum Nahn Jee.

**Investigation:** Keum Nahn Jee.

**Project administration:** Keum Nahn Jee.

**Supervision:** Keum Nahn Jee.

**Validation:** Keum Nahn Jee.

**Writing – original draft:** Keum Nahn Jee.

**Writing – review & editing:** Keum Nahn Jee.
